# Poly[[aqua­[μ_5_-5-(isonicotinamido)­isophthalato][μ_4_-5-(isonicotinamido)­isophthalato]holmium(III)silver(I)] dihydrate]

**DOI:** 10.1107/S1600536812028620

**Published:** 2012-06-30

**Authors:** Xue Nie, Jing-Nian Qu

**Affiliations:** a Department of Chemistry and Materials Science, Hengyang Normal University, Hengyang Hunan 421008, People’s Republic of China

## Abstract

The title heteronuclear complex, {[AgHo(C_14_H_8_N_2_O_5_)_2_(H_2_O)]·2H_2_O}_*n*_, has a three-dimensional polymeric structure, generated by the carboxyl­ate and pyridine groups of the 5-(isonicotinamido)­isophthalate (INAIP) ligands bridging the metal atoms. The Ho^III^ atom is coordinated by seven O atoms from INAIP ligands and a water mol­ecule in a distorted square-anti­prismatic geometry, while the Ag^I^ atom has a distorted trigonal-planar AgN_2_O geometry. Inter­molecular O—H⋯O and N—H⋯O hydrogen bonds stabilize the crystal structure.

## Related literature

For background to coordination polymeric frameworks, see: Kapoor *et al.* (2002 ▶); Abourahma *et al.* (2002 ▶); Costes *et al.* (2004 ▶). For related hetero-metallic complexes, see: Chen *et al.* (2010 ▶); Liang *et al.* (2000 ▶); Zhao *et al.* (2003 ▶, 2004 ▶); Nie & Qu (2011 ▶); Zhang *et al.* (2005 ▶); Cheng *et al.* (2006 ▶); Lin *et al.* (2009 ▶).
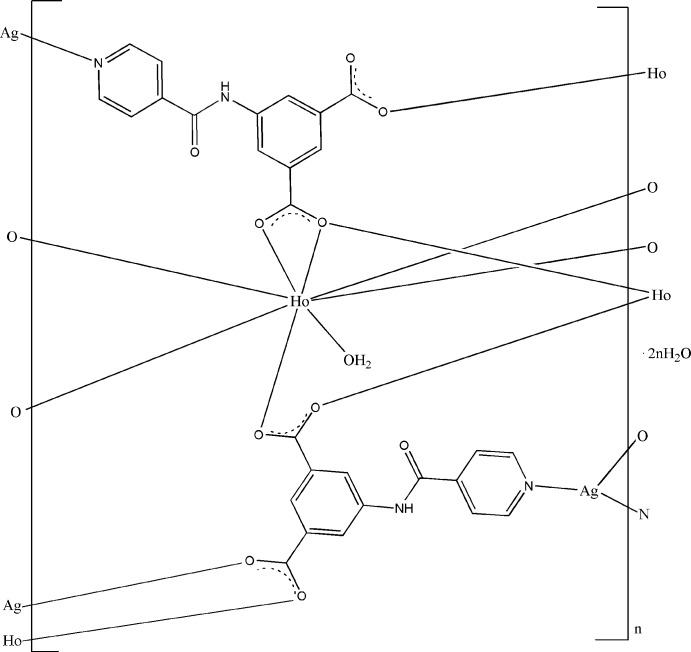


## Experimental

### 

#### Crystal data

[AgHo(C_14_H_8_N_2_O_5_)_2_(H_2_O)]·2H_2_O
*M* *_r_* = 895.30
Triclinic, 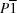

*a* = 9.8343 (13) Å
*b* = 11.3087 (15) Å
*c* = 13.723 (2) Å
α = 73.914 (2)°
β = 70.671 (1)°
γ = 83.965 (2)°
*V* = 1383.6 (3) Å^3^
*Z* = 2
Mo *K*α radiation
μ = 3.63 mm^−1^
*T* = 291 K
0.20 × 0.16 × 0.10 mm

#### Data collection

Bruker SMART APEXII CCD diffractometer
Absorption correction: multi-scan (*SADABS*; Bruker, 2001 ▶) *T*_min_ = 0.531, *T*_max_ = 0.713
7445 measured reflections
5273 independent reflections
4177 reflections with *I* > 2σ(*I*)
*R*_int_ = 0.024

#### Refinement

*R*[*F* ^2^ > 2σ(*F* ^2^)] = 0.040
*wR*(*F* ^2^) = 0.104
*S* = 1.03
5273 reflections
424 parameters
2 restraints
H-atom parameters constrained
Δρ_max_ = 1.37 e Å^−3^
Δρ_min_ = −1.56 e Å^−3^

### 

Data collection: *APEX2* (Bruker, 2007 ▶); cell refinement: *SAINT* (Bruker, 2007 ▶); data reduction: *SAINT*; program(s) used to solve structure: *SHELXTL* (Sheldrick, 2008 ▶); program(s) used to refine structure: *SHELXTL*; molecular graphics: *SHELXTL*; software used to prepare material for publication: *SHELXTL*.

## Supplementary Material

Crystal structure: contains datablock(s) global, I. DOI: 10.1107/S1600536812028620/xu5562sup1.cif

Structure factors: contains datablock(s) I. DOI: 10.1107/S1600536812028620/xu5562Isup2.hkl

Additional supplementary materials:  crystallographic information; 3D view; checkCIF report

## Figures and Tables

**Table 1:** Hydrogen-bond geometry (Å, °)

*D*—H⋯*A*	*D*—H	H⋯*A*	*D*⋯*A*	*D*—H⋯*A*
N2—H2⋯O3*W* ^i^	0.86	2.11	2.878 (9)	149
N4—H4⋯O5^ii^	0.86	2.08	2.925 (8)	168
O1*W*—H1*X*⋯O9^iii^	0.85	2.05	2.562 (7)	118
O1*W*—H1*Y*⋯O10^iv^	0.85	1.90	2.717 (7)	161
O2*W*—H2*X*⋯O3*W* ^v^	0.85	2.11	2.799 (8)	138
O2*W*—H2*Y*⋯O1*W* ^vi^	0.85	2.34	3.136 (8)	156
O2*W*—H2*Y*⋯O9^vii^	0.85	2.22	2.766 (8)	122
O3*W*—H3*X*⋯N2^viii^	0.85	2.49	3.187 (9)	140
O3*W*—H3*Y*⋯O9^ix^	0.85	2.30	2.821 (7)	120

Symmetry codes: (i) 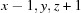; (ii) 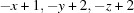; (iii) 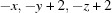; (iv) 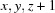; (v) 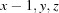; (vi) 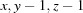; (vii) 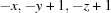; (viii) 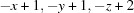; (ix) 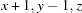.

**Fig. 1.**
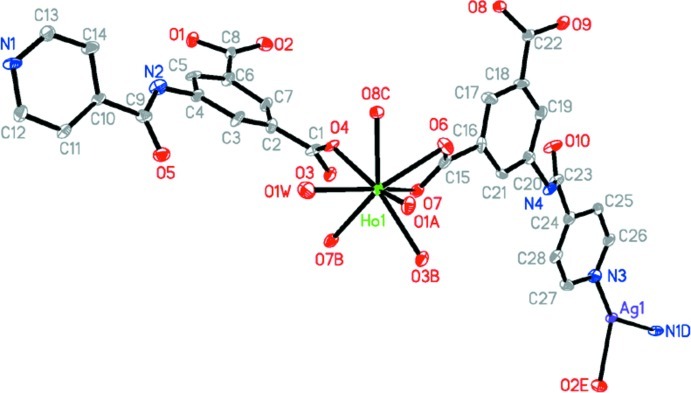
The ORTEP drawing of the title compound (I). Displacement ellipsoids are drawn at 30% probability level.

**Fig. 2.**
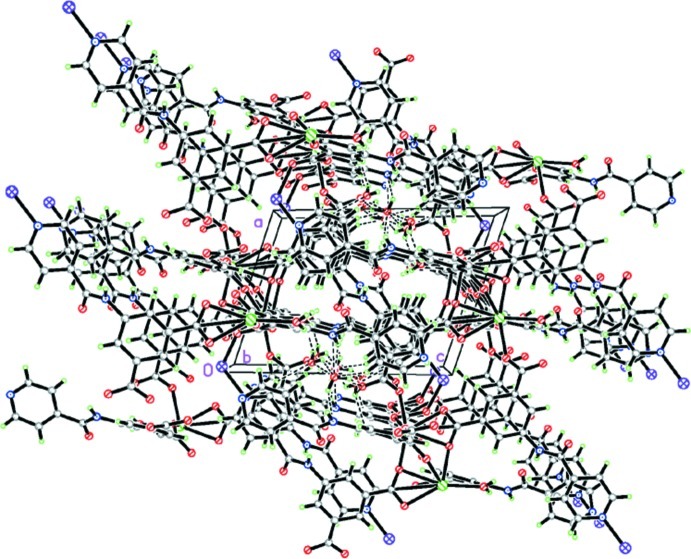
Projection showing the three-dimensional structure of the compound (I).

## supplementary crystallographic information

### Comment

Recently, coordination polymeric frameworks have attracted great attention due to their potential applications and intriguing structure topologies (Kapoor *et al.*, 2002; Abourahma *et al.*, 2002; Costes *et al.*, 2004). However, to obtain d-f coordination polymers is more important. On the other hand, multidentate ligands containing both N– and O-donor atoms are usually employed in the construction of Lanthanide and transiton metal heterometallic structures, in keeping with the typical coordination behaviors of Ln and *M* ions under different reaction conditions (Zhang *et al.*, 2005; Cheng *et al.*, 2006; Lin *et al.*, 2009). To the best of our knowledge, 5-(isonicotinamido)isophthalic acid (H_2_INAIP) can show richer coordination modes due to its two carboxylate groups and one pyridyl group, accordingly, it is an excellent candidate for the construction of metal organic frameworks (Chen *et al.*, 2010). In this paper, we report on the synthesis and crystal structure of a 4d-4f heterometallic coordination polymer (I).

It is interesting that two INAIP^2-^ ligands exhibit different coordination modes: one coordinated to three Ho^III^ atoms and two Ag^I^ atoms while the other coordinated to three Ho^III^ atoms and one Ag^I^ atom, originated from the different coordination modes of the carboxylate groups. When the Ag—N and Ag—O connections are neglected, a two-dimensional (4,4) bilayer network is formed by Ho(III)-carboxylate groups, which is similar complex [AgCe(C_14_H_8_N_2_O_5_)_2_(H_2_O)]_n_ (Nie *et al.*, 2011). Then the two-dimensional (4,4) nets are linked together by Ag—N and Ag—O coordination interaction to form a complicated three-dimensional supramolecular net (Fig. 2), which is isomorphous to its AgEr isologue {[AgEr(INAIP)_2_(H_2_O)].H_2_O}_n_ (Chen *et al.*, 2010).

### Experimental

A mixture of 0.05 mmol Ho(NO_3_)_3_.6H_2_O (23.0 mg, 0.05 mmol), H_2_INAIP (28.6 mg, 0.1 mmol), AgNO_3_ (8.5 mg, 0.05 mmol), NaOH (6.0 mg, 0.15 mmol) and H_2_O (10 ml) was heated in a 16 mL capacity Teflon-lined reaction vessel at 453 K for 4 d, the reaction mixture was cooled to room temperature over a period of 40 h. The product was collected by filtration.

### Refinement

H atoms bonded to C atoms were placed geometrically and refined as riding atoms. The pyridyl N atoms were found from a difference Fourier maps and refined as riding with N—H = 0.86 Å, and the water H atoms were found from Fourier difference maps and refined with restraints for O—H distances 0.85 Å with *U*_iso_(H) = 1.2*U*_eq_(O).

### Crystal data

**Table:**

[AgHo(C_14_H_8_N_2_O_5_)_2_(H_2_O)]·2H_2_O	*Z* = 2
*M**_r_* = 895.30	*F*(000) = 872
Triclinic, *P*1	*D*_x_ = 2.149 Mg m^−^^3^
Hall symbol: -P 1	Mo *K*α radiation, λ = 0.71073 Å
*a* = 9.8343 (13) Å	Cell parameters from 3132 reflections
*b* = 11.3087 (15) Å	θ = 2.1–25.3°
*c* = 13.723 (2) Å	µ = 3.63 mm^−^^1^
α = 73.914 (2)°	*T* = 291 K
β = 70.671 (1)°	Block, colorless
γ = 83.965 (2)°	0.20 × 0.16 × 0.10 mm
*V* = 1383.6 (3) Å^3^	

### Data collection

**Table:**

Bruker SMART APEXII CCD diffractometer	5273 independent reflections
Radiation source: sealed tube	4177 reflections with *I* > 2σ(*I*)
Graphite monochromator	*R*_int_ = 0.024
φ and ω scans	θ_max_ = 26.0°, θ_min_ = 2.1°
Absorption correction: multi-scan (*SADABS*; Bruker, 2001)	*h* = −12→10
*T*_min_ = 0.531, *T*_max_ = 0.713	*k* = −13→12
7445 measured reflections	*l* = −16→12

### Refinement

**Table:**

Refinement on *F*^2^	Primary atom site location: structure-invariant direct methods
Least-squares matrix: full	Secondary atom site location: difference Fourier map
*R*[*F*^2^ > 2σ(*F*^2^)] = 0.040	Hydrogen site location: inferred from neighbouring sites
*wR*(*F*^2^) = 0.104	H-atom parameters constrained
*S* = 1.03	*w* = 1/[σ^2^(*F*_o_^2^) + (0.065*P*)^2^ + 1.99*P*] where *P* = (*F*_o_^2^ + 2*F*_c_^2^)/3
5273 reflections	(Δ/σ)_max_ < 0.001
424 parameters	Δρ_max_ = 1.37 e Å^−^^3^
2 restraints	Δρ_min_ = −1.56 e Å^−^^3^

### Special details

**Table:**

Geometry. All e.s.d.'s (except the e.s.d. in the dihedral angle between two l.s. planes) are estimated using the full covariance matrix. The cell e.s.d.'s are taken into account individually in the estimation of e.s.d.'s in distances, angles and torsion angles; correlations between e.s.d.'s in cell parameters are only used when they are defined by crystal symmetry. An approximate (isotropic) treatment of cell e.s.d.'s is used for estimating e.s.d.'s involving l.s. planes.
Refinement. Refinement of *F*^2^ against ALL reflections. The weighted *R*-factor *wR* and goodness of fit *S* are based on *F*^2^, conventional *R*-factors *R* are based on *F*, with *F* set to zero for negative *F*^2^. The threshold expression of *F*^2^ > σ(*F*^2^) is used only for calculating *R*-factors(gt) *etc*. and is not relevant to the choice of reflections for refinement. *R*-factors based on *F*^2^ are statistically about twice as large as those based on *F*, and *R*- factors based on ALL data will be even larger.

### Fractional atomic coordinates and isotropic or equivalent isotropic displacement parameters (Å^2^)

**Table:**

	*x*	*y*	*z*	*U*_iso_*/*U*_eq_	
Ag1	1.04960 (5)	1.30998 (4)	0.01447 (3)	0.02214 (13)	
N1	0.1196 (5)	0.3945 (5)	1.8413 (4)	0.0258 (12)	
C1	0.3868 (7)	0.7756 (6)	1.0898 (5)	0.0294 (14)	
C2	0.3587 (7)	0.6471 (6)	1.1524 (5)	0.0251 (13)	
C3	0.3163 (8)	0.6261 (6)	1.2659 (6)	0.0308 (15)	
H3	0.3088	0.6906	1.2974	0.037*	
C4	0.2868 (7)	0.5064 (6)	1.3275 (5)	0.0270 (14)	
C5	0.2826 (7)	0.4137 (7)	1.2831 (6)	0.0300 (15)	
H5	0.2614	0.3345	1.3269	0.036*	
C6	0.3089 (7)	0.4344 (6)	1.1751 (5)	0.0275 (14)	
C7	0.3509 (7)	0.5518 (6)	1.1080 (6)	0.0287 (15)	
H7	0.3734	0.5662	1.0344	0.034*	
C8	0.2794 (7)	0.3470 (6)	1.1205 (5)	0.0236 (13)	
C9	0.2932 (7)	0.5305 (6)	1.4989 (4)	0.0264 (14)	
C10	0.2362 (6)	0.4795 (5)	1.6187 (4)	0.0205 (12)	
C11	0.3136 (7)	0.4793 (6)	1.6833 (5)	0.0261 (13)	
H11	0.4068	0.5096	1.6541	0.031*	
C12	0.2550 (8)	0.4341 (6)	1.7940 (5)	0.0289 (14)	
H12	0.3125	0.4313	1.8364	0.035*	
C13	0.0468 (8)	0.3953 (6)	1.7772 (5)	0.0292 (14)	
H13	−0.0463	0.3649	1.8087	0.035*	
C14	0.0931 (7)	0.4361 (7)	1.6693 (5)	0.0317 (15)	
H14	0.0329	0.4357	1.6295	0.038*	
C15	0.3121 (8)	1.0833 (6)	0.9041 (5)	0.0316 (15)	
C16	0.2768 (8)	1.1040 (6)	0.8032 (5)	0.0293 (14)	
C17	0.1427 (7)	1.0716 (7)	0.8095 (5)	0.0309 (15)	
H17	0.0730	1.0425	0.8752	0.037*	
C18	0.1136 (7)	1.0844 (7)	0.7096 (5)	0.0281 (14)	
C19	0.2151 (8)	1.1266 (6)	0.6153 (5)	0.0282 (14)	
H19	0.1951	1.1397	0.5515	0.034*	
C20	0.3512 (7)	1.1504 (6)	0.6155 (5)	0.0259 (14)	
C21	0.3801 (6)	1.1449 (6)	0.7074 (5)	0.0211 (12)	
H21	0.4693	1.1688	0.7046	0.025*	
C22	−0.0289 (7)	1.0459 (5)	0.7172 (5)	0.0238 (13)	
C23	0.4874 (7)	1.1543 (6)	0.4272 (5)	0.0263 (14)	
C24	0.6258 (7)	1.1979 (6)	0.3365 (5)	0.0261 (13)	
C25	0.6156 (7)	1.2202 (6)	0.2338 (5)	0.0245 (13)	
H25	0.5284	1.2121	0.2238	0.029*	
C26	0.7390 (7)	1.2549 (6)	0.1470 (6)	0.0292 (14)	
H26	0.7324	1.2708	0.0785	0.035*	
C27	0.8703 (7)	1.2411 (7)	0.2579 (5)	0.0287 (14)	
H27	0.9591	1.2431	0.2677	0.034*	
C28	0.7498 (7)	1.2132 (6)	0.3449 (5)	0.0276 (14)	
H28	0.7569	1.2049	0.4123	0.033*	
Ho1	0.34584 (3)	1.06199 (3)	1.10929 (2)	0.02108 (10)	
N2	0.2485 (7)	0.4741 (6)	1.4411 (5)	0.0308 (13)	
H2	0.1916	0.4130	1.4757	0.037*	
N3	0.8624 (6)	1.2658 (6)	0.1574 (4)	0.0304 (13)	
N4	0.4619 (6)	1.1984 (5)	0.5160 (4)	0.0255 (11)	
H4	0.5143	1.2571	0.5118	0.031*	
O1	0.2805 (5)	0.2273 (4)	1.1729 (4)	0.0288 (10)	
O2	0.2510 (5)	0.3806 (5)	1.0383 (4)	0.0331 (11)	
O3	0.4852 (5)	0.7904 (4)	1.0006 (4)	0.0266 (10)	
O4	0.3161 (5)	0.8618 (4)	1.1245 (3)	0.0243 (9)	
O5	0.3783 (5)	0.6113 (5)	1.4626 (4)	0.0315 (11)	
O6	0.2220 (5)	1.1194 (5)	0.9793 (4)	0.0299 (10)	
O7	0.4297 (5)	1.0367 (4)	0.9108 (4)	0.0260 (10)	
O8	−0.1050 (5)	0.9720 (4)	0.8033 (4)	0.0262 (10)	
O9	−0.0714 (5)	1.0863 (5)	0.6377 (4)	0.0326 (11)	
O10	0.4053 (6)	1.0890 (5)	0.4210 (3)	0.0355 (12)	
O1W	0.3331 (5)	0.9853 (5)	1.2887 (4)	0.0379 (12)	
H1X	0.2912	0.9176	1.3251	0.045*	
H1Y	0.3707	1.0249	1.3174	0.045*	
O2W	0.0736 (6)	0.1475 (5)	0.3881 (5)	0.0429 (13)	
H2X	0.0917	0.1841	0.4287	0.052*	
H2Y	0.1332	0.0882	0.3796	0.052*	
O3W	0.9981 (5)	0.3256 (4)	0.5043 (4)	0.0349 (12)	
H3X	0.9205	0.3620	0.4971	0.042*	
H3Y	0.9858	0.2911	0.5700	0.042*	

### Atomic displacement parameters (Å^2^)

**Table:**

	*U*^11^	*U*^22^	*U*^33^	*U*^12^	*U*^13^	*U*^23^
Ag1	0.0179 (2)	0.0271 (3)	0.0160 (2)	−0.00653 (18)	0.00087 (17)	−0.00215 (18)
N1	0.015 (2)	0.039 (3)	0.021 (3)	−0.007 (2)	0.006 (2)	−0.015 (2)
C1	0.024 (3)	0.031 (4)	0.027 (3)	−0.012 (3)	0.002 (3)	−0.005 (3)
C2	0.030 (3)	0.018 (3)	0.022 (3)	0.004 (2)	−0.006 (3)	−0.003 (2)
C3	0.044 (4)	0.010 (3)	0.034 (4)	−0.010 (3)	−0.008 (3)	−0.001 (3)
C4	0.030 (3)	0.034 (4)	0.014 (3)	−0.012 (3)	−0.006 (3)	0.001 (3)
C5	0.016 (3)	0.033 (4)	0.034 (4)	−0.012 (3)	0.002 (3)	−0.005 (3)
C6	0.035 (4)	0.027 (4)	0.017 (3)	−0.005 (3)	−0.003 (3)	−0.005 (3)
C7	0.024 (3)	0.015 (3)	0.036 (4)	0.002 (2)	−0.003 (3)	0.002 (3)
C8	0.020 (3)	0.027 (3)	0.018 (3)	−0.004 (2)	−0.002 (2)	−0.001 (2)
C9	0.030 (3)	0.026 (3)	0.030 (3)	−0.006 (3)	−0.012 (3)	−0.013 (3)
C10	0.026 (3)	0.012 (3)	0.026 (3)	−0.007 (2)	−0.007 (3)	−0.005 (2)
C11	0.030 (3)	0.019 (3)	0.025 (3)	−0.008 (2)	−0.004 (3)	−0.001 (2)
C12	0.037 (4)	0.026 (3)	0.020 (3)	−0.005 (3)	−0.007 (3)	−0.002 (3)
C13	0.033 (4)	0.033 (4)	0.023 (3)	−0.008 (3)	−0.010 (3)	−0.005 (3)
C14	0.023 (3)	0.044 (4)	0.023 (3)	−0.011 (3)	0.002 (3)	−0.008 (3)
C15	0.031 (4)	0.025 (4)	0.024 (3)	−0.008 (3)	0.004 (3)	0.004 (3)
C16	0.039 (4)	0.024 (3)	0.020 (3)	0.005 (3)	−0.012 (3)	0.003 (2)
C17	0.016 (3)	0.053 (5)	0.018 (3)	−0.003 (3)	0.002 (2)	−0.007 (3)
C18	0.014 (3)	0.039 (4)	0.026 (3)	−0.007 (3)	0.003 (2)	−0.006 (3)
C19	0.045 (4)	0.018 (3)	0.027 (3)	−0.007 (3)	−0.018 (3)	−0.003 (2)
C20	0.020 (3)	0.020 (3)	0.032 (3)	0.009 (2)	−0.004 (3)	−0.007 (3)
C21	0.018 (3)	0.021 (3)	0.029 (3)	−0.002 (2)	−0.013 (2)	−0.005 (2)
C22	0.020 (3)	0.011 (3)	0.034 (3)	−0.001 (2)	−0.001 (3)	−0.006 (2)
C23	0.031 (3)	0.026 (3)	0.020 (3)	−0.009 (3)	0.000 (3)	−0.009 (3)
C24	0.029 (3)	0.019 (3)	0.028 (3)	0.000 (2)	−0.003 (3)	−0.007 (3)
C25	0.023 (3)	0.017 (3)	0.032 (3)	0.005 (2)	−0.009 (3)	−0.006 (2)
C26	0.026 (3)	0.024 (3)	0.028 (3)	−0.002 (3)	0.000 (3)	−0.001 (3)
C27	0.020 (3)	0.041 (4)	0.024 (3)	−0.004 (3)	−0.003 (3)	−0.009 (3)
C28	0.032 (4)	0.019 (3)	0.024 (3)	−0.003 (3)	0.001 (3)	−0.005 (2)
Ho1	0.01787 (16)	0.02171 (16)	0.01800 (15)	−0.00186 (10)	−0.00121 (11)	−0.00102 (10)
N2	0.037 (3)	0.033 (3)	0.024 (3)	−0.008 (3)	−0.003 (2)	−0.015 (2)
N3	0.030 (3)	0.039 (3)	0.016 (3)	−0.006 (2)	0.001 (2)	−0.003 (2)
N4	0.029 (3)	0.027 (3)	0.014 (2)	−0.012 (2)	0.001 (2)	0.000 (2)
O1	0.030 (2)	0.027 (2)	0.020 (2)	0.0043 (19)	−0.0004 (19)	−0.0024 (18)
O2	0.031 (3)	0.046 (3)	0.030 (3)	−0.003 (2)	−0.012 (2)	−0.018 (2)
O3	0.025 (2)	0.021 (2)	0.025 (2)	0.0010 (18)	−0.0009 (19)	0.0004 (17)
O4	0.021 (2)	0.023 (2)	0.020 (2)	−0.0115 (18)	0.0050 (17)	−0.0019 (17)
O5	0.031 (3)	0.034 (3)	0.030 (3)	−0.010 (2)	−0.009 (2)	−0.007 (2)
O6	0.028 (2)	0.045 (3)	0.020 (2)	0.010 (2)	−0.0083 (19)	−0.016 (2)
O7	0.020 (2)	0.019 (2)	0.029 (2)	0.0000 (17)	0.0020 (18)	−0.0033 (18)
O8	0.018 (2)	0.030 (2)	0.025 (2)	−0.0024 (18)	−0.0029 (18)	−0.0022 (19)
O9	0.033 (3)	0.037 (3)	0.018 (2)	−0.010 (2)	−0.0044 (19)	0.0075 (19)
O10	0.044 (3)	0.048 (3)	0.014 (2)	−0.028 (3)	0.004 (2)	−0.012 (2)
O1W	0.030 (3)	0.054 (3)	0.027 (3)	−0.002 (2)	−0.007 (2)	−0.007 (2)
O2W	0.046 (3)	0.032 (3)	0.044 (3)	−0.015 (2)	−0.016 (3)	0.006 (2)
O3W	0.033 (3)	0.029 (3)	0.037 (3)	−0.010 (2)	−0.012 (2)	0.006 (2)

### Geometric parameters (Å, º)

**Table:**

Ag1—N3	2.178 (5)	C18—C22	1.473 (9)
Ag1—N1^i^	2.198 (5)	C19—C20	1.393 (10)
Ag1—O2^ii^	2.361 (5)	C19—H19	0.9300
N1—C13	1.304 (8)	C20—C21	1.366 (9)
N1—C12	1.337 (9)	C20—N4	1.440 (8)
N1—Ag1^iii^	2.198 (5)	C21—H21	0.9300
C1—O4	1.248 (9)	C22—O9	1.249 (8)
C1—O3	1.265 (8)	C22—O8	1.285 (8)
C1—C2	1.469 (9)	C23—O10	1.187 (8)
C2—C7	1.396 (10)	C23—N4	1.381 (8)
C2—C3	1.430 (10)	C23—C24	1.524 (9)
C3—C4	1.385 (9)	C24—C28	1.295 (10)
C3—H3	0.9300	C24—C25	1.398 (9)
C4—C5	1.360 (10)	C25—C26	1.389 (9)
C4—N2	1.425 (8)	C25—H25	0.9300
C5—C6	1.373 (10)	C26—N3	1.290 (9)
C5—H5	0.9300	C26—H26	0.9300
C6—C7	1.402 (9)	C27—N3	1.357 (9)
C6—C8	1.496 (9)	C27—C28	1.364 (9)
C7—H7	0.9300	C27—H27	0.9300
C8—O2	1.201 (8)	C28—H28	0.9300
C8—O1	1.347 (8)	Ho1—O1^iv^	2.225 (5)
C9—O5	1.190 (7)	Ho1—O4	2.256 (4)
C9—N2	1.342 (8)	Ho1—O8^v^	2.291 (4)
C9—C10	1.5102 (11)	Ho1—O3^vi^	2.313 (4)
C10—C11	1.347 (9)	Ho1—O7^vi^	2.333 (4)
C10—C14	1.418 (9)	Ho1—O1W	2.337 (5)
C11—C12	1.397 (9)	Ho1—O6	2.391 (4)
C11—H11	0.9300	Ho1—O7	2.660 (5)
C12—H12	0.9300	N2—H2	0.8600
C13—C14	1.353 (9)	N4—H4	0.8600
C13—H13	0.9300	O1—Ho1^vii^	2.225 (5)
C14—H14	0.9300	O2—Ag1^viii^	2.361 (5)
C15—O7	1.241 (9)	O3—Ho1^vi^	2.313 (4)
C15—O6	1.252 (9)	O7—Ho1^vi^	2.333 (4)
C15—C16	1.488 (10)	O8—Ho1^v^	2.291 (4)
C15—Ho1	2.885 (7)	O1W—H1X	0.8501
C16—C21	1.361 (9)	O1W—H1Y	0.8500
C16—C17	1.375 (10)	O2W—H2X	0.8500
C17—C18	1.457 (9)	O2W—H2Y	0.8500
C17—H17	0.9300	O3W—H3X	0.8500
C18—C19	1.342 (9)	O3W—H3Y	0.8500
			
N3—Ag1—N1^i^	144.2 (2)	C26—C25—C24	118.3 (6)
N3—Ag1—O2^ii^	115.2 (2)	C26—C25—H25	120.8
N1^i^—Ag1—O2^ii^	93.50 (18)	C24—C25—H25	120.8
C13—N1—C12	115.7 (6)	N3—C26—C25	122.8 (7)
C13—N1—Ag1^iii^	125.4 (4)	N3—C26—H26	118.6
C12—N1—Ag1^iii^	118.3 (4)	C25—C26—H26	118.6
O4—C1—O3	123.9 (6)	N3—C27—C28	120.9 (6)
O4—C1—C2	120.8 (6)	N3—C27—H27	119.5
O3—C1—C2	115.3 (6)	C28—C27—H27	119.5
C7—C2—C3	119.8 (6)	C24—C28—C27	122.2 (7)
C7—C2—C1	123.4 (6)	C24—C28—H28	118.9
C3—C2—C1	116.1 (6)	C27—C28—H28	118.9
C4—C3—C2	117.7 (6)	O1^iv^—Ho1—O4	149.70 (16)
C4—C3—H3	121.2	O1^iv^—Ho1—O8^v^	78.68 (18)
C2—C3—H3	121.2	O4—Ho1—O8^v^	75.05 (16)
C5—C4—C3	121.5 (6)	O1^iv^—Ho1—O3^vi^	74.53 (17)
C5—C4—N2	115.8 (6)	O4—Ho1—O3^vi^	135.51 (16)
C3—C4—N2	122.5 (6)	O8^v^—Ho1—O3^vi^	144.42 (16)
C4—C5—C6	121.5 (6)	O1^iv^—Ho1—O7^vi^	124.68 (18)
C4—C5—H5	119.3	O4—Ho1—O7^vi^	72.06 (16)
C6—C5—H5	119.3	O8^v^—Ho1—O7^vi^	141.58 (16)
C5—C6—C7	119.3 (6)	O3^vi^—Ho1—O7^vi^	73.90 (16)
C5—C6—C8	125.9 (6)	O1^iv^—Ho1—O1W	77.24 (18)
C7—C6—C8	114.3 (6)	O4—Ho1—O1W	82.04 (18)
C2—C7—C6	119.6 (6)	O8^v^—Ho1—O1W	76.21 (17)
C2—C7—H7	120.2	O3^vi^—Ho1—O1W	119.05 (18)
C6—C7—H7	120.2	O7^vi^—Ho1—O1W	80.08 (17)
O2—C8—O1	122.1 (6)	O1^iv^—Ho1—O6	96.32 (17)
O2—C8—C6	122.9 (6)	O4—Ho1—O6	89.69 (17)
O1—C8—C6	114.9 (5)	O8^v^—Ho1—O6	71.91 (16)
O5—C9—N2	124.8 (5)	O3^vi^—Ho1—O6	88.12 (17)
O5—C9—C10	119.3 (5)	O7^vi^—Ho1—O6	126.46 (16)
N2—C9—C10	115.8 (5)	O1W—Ho1—O6	148.13 (17)
C11—C10—C14	116.5 (5)	O1^iv^—Ho1—O7	131.53 (15)
C11—C10—C9	122.5 (5)	O4—Ho1—O7	73.93 (14)
C14—C10—C9	120.9 (5)	O8^v^—Ho1—O7	113.37 (15)
C10—C11—C12	120.5 (6)	O3^vi^—Ho1—O7	70.57 (16)
C10—C11—H11	119.7	O7^vi^—Ho1—O7	75.75 (17)
C12—C11—H11	119.7	O1W—Ho1—O7	150.06 (17)
N1—C12—C11	122.7 (6)	O6—Ho1—O7	50.74 (15)
N1—C12—H12	118.7	O1^iv^—Ho1—C15	115.62 (19)
C11—C12—H12	118.7	O4—Ho1—C15	80.58 (18)
N1—C13—C14	126.5 (7)	O8^v^—Ho1—C15	92.40 (18)
N1—C13—H13	116.8	O3^vi^—Ho1—C15	78.82 (18)
C14—C13—H13	116.8	O7^vi^—Ho1—C15	101.17 (19)
C13—C14—C10	118.1 (6)	O1W—Ho1—C15	161.2 (2)
C13—C14—H14	121.0	O6—Ho1—C15	25.30 (18)
C10—C14—H14	121.0	O7—Ho1—C15	25.45 (17)
O7—C15—O6	121.8 (7)	O1^iv^—Ho1—Ho1^vi^	141.56 (12)
O7—C15—C16	120.5 (6)	O4—Ho1—Ho1^vi^	68.37 (11)
O6—C15—C16	117.7 (7)	O8^v^—Ho1—Ho1^vi^	136.93 (12)
O7—C15—Ho1	67.1 (4)	O3^vi^—Ho1—Ho1^vi^	67.16 (12)
O6—C15—Ho1	54.7 (4)	O7^vi^—Ho1—Ho1^vi^	40.79 (12)
C16—C15—Ho1	172.3 (5)	O1W—Ho1—Ho1^vi^	118.83 (13)
C21—C16—C17	121.6 (6)	O6—Ho1—Ho1^vi^	85.69 (11)
C21—C16—C15	119.8 (6)	O7—Ho1—Ho1^vi^	34.96 (10)
C17—C16—C15	118.4 (6)	C15—Ho1—Ho1^vi^	60.39 (15)
C16—C17—C18	117.6 (6)	C9—N2—C4	125.9 (6)
C16—C17—H17	121.2	C9—N2—H2	117.1
C18—C17—H17	121.2	C4—N2—H2	117.1
C19—C18—C17	120.6 (6)	C26—N3—C27	117.8 (6)
C19—C18—C22	122.2 (6)	C26—N3—Ag1	118.9 (5)
C17—C18—C22	117.2 (6)	C27—N3—Ag1	123.2 (5)
C18—C19—C20	118.4 (6)	C23—N4—C20	122.8 (6)
C18—C19—H19	120.8	C23—N4—H4	118.6
C20—C19—H19	120.8	C20—N4—H4	118.6
C21—C20—C19	122.4 (6)	C8—O1—Ho1^vii^	129.9 (4)
C21—C20—N4	117.1 (6)	C8—O2—Ag1^viii^	123.1 (5)
C19—C20—N4	120.0 (6)	C1—O3—Ho1^vi^	137.0 (4)
C16—C21—C20	119.2 (6)	C1—O4—Ho1	138.4 (4)
C16—C21—H21	120.4	C15—O6—Ho1	100.0 (4)
C20—C21—H21	120.4	C15—O7—Ho1^vi^	167.9 (5)
O9—C22—O8	121.9 (6)	C15—O7—Ho1	87.5 (4)
O9—C22—C18	117.9 (6)	Ho1^vi^—O7—Ho1	104.25 (17)
O8—C22—C18	120.3 (6)	C22—O8—Ho1^v^	136.1 (4)
O10—C23—N4	122.2 (6)	Ho1—O1W—H1X	120.0
O10—C23—C24	122.3 (6)	Ho1—O1W—H1Y	120.0
N4—C23—C24	115.5 (6)	H1X—O1W—H1Y	120.0
C28—C24—C25	117.7 (6)	H2X—O2W—H2Y	109.5
C28—C24—C23	127.3 (6)	H3X—O3W—H3Y	109.5
C25—C24—C23	115.0 (6)		
			
O4—C1—C2—C7	136.4 (7)	O6—C15—Ho1—O7^vi^	179.2 (4)
O3—C1—C2—C7	−43.4 (9)	O7—C15—Ho1—O1W	−94.8 (7)
O4—C1—C2—C3	−33.9 (10)	O6—C15—Ho1—O1W	87.2 (7)
O3—C1—C2—C3	146.3 (7)	O7—C15—Ho1—O6	178.0 (7)
C7—C2—C3—C4	8.3 (10)	O6—C15—Ho1—O7	−178.0 (7)
C1—C2—C3—C4	179.0 (6)	O7—C15—Ho1—Ho1^vi^	−1.9 (3)
C2—C3—C4—C5	−7.0 (11)	O6—C15—Ho1—Ho1^vi^	−179.8 (5)
C2—C3—C4—N2	177.8 (6)	C10—C9—N2—C4	179.5 (6)
C3—C4—C5—C6	0.7 (11)	C5—C4—N2—C9	153.6 (7)
N2—C4—C5—C6	176.2 (6)	C3—C4—N2—C9	−31.0 (11)
C4—C5—C6—C7	4.5 (11)	C25—C26—N3—C27	0.6 (11)
C4—C5—C6—C8	−167.2 (7)	C25—C26—N3—Ag1	176.3 (5)
C3—C2—C7—C6	−3.4 (10)	C28—C27—N3—C26	−4.1 (11)
C1—C2—C7—C6	−173.4 (6)	C28—C27—N3—Ag1	−179.6 (5)
C5—C6—C7—C2	−3.0 (10)	N1^i^—Ag1—N3—C26	23.8 (8)
C8—C6—C7—C2	169.6 (6)	O2^ii^—Ag1—N3—C26	163.9 (5)
C5—C6—C8—O2	150.6 (7)	N1^i^—Ag1—N3—C27	−160.7 (5)
C7—C6—C8—O2	−21.4 (10)	O2^ii^—Ag1—N3—C27	−20.6 (6)
C5—C6—C8—O1	−26.4 (10)	O10—C23—N4—C20	12.9 (11)
C7—C6—C8—O1	161.5 (6)	C24—C23—N4—C20	−168.7 (6)
O5—C9—C10—C11	−29.0 (10)	C21—C20—N4—C23	144.5 (7)
N2—C9—C10—C11	147.1 (6)	C19—C20—N4—C23	−43.6 (9)
O5—C9—C10—C14	146.9 (7)	O2—C8—O1—Ho1^vii^	35.2 (9)
N2—C9—C10—C14	−37.0 (9)	C6—C8—O1—Ho1^vii^	−147.7 (5)
C14—C10—C11—C12	2.2 (9)	O1—C8—O2—Ag1^viii^	47.6 (8)
C9—C10—C11—C12	178.3 (6)	C6—C8—O2—Ag1^viii^	−129.2 (6)
C13—N1—C12—C11	3.1 (10)	O4—C1—O3—Ho1^vi^	30.8 (11)
Ag1^iii^—N1—C12—C11	174.4 (5)	C2—C1—O3—Ho1^vi^	−149.4 (5)
C10—C11—C12—N1	−3.1 (11)	O3—C1—O4—Ho1	−28.5 (11)
C12—N1—C13—C14	−2.6 (11)	C2—C1—O4—Ho1	151.7 (5)
Ag1^iii^—N1—C13—C14	−173.2 (6)	O1^iv^—Ho1—O4—C1	−159.5 (6)
N1—C13—C14—C10	2.0 (12)	O8^v^—Ho1—O4—C1	169.8 (7)
C11—C10—C14—C13	−1.7 (10)	O3^vi^—Ho1—O4—C1	11.4 (8)
C9—C10—C14—C13	−177.8 (6)	O7^vi^—Ho1—O4—C1	−30.3 (7)
O7—C15—C16—C21	−39.9 (10)	O1W—Ho1—O4—C1	−112.4 (7)
O6—C15—C16—C21	137.0 (7)	O6—Ho1—O4—C1	98.5 (7)
O7—C15—C16—C17	135.0 (7)	O7—Ho1—O4—C1	49.5 (7)
O6—C15—C16—C17	−48.2 (10)	C15—Ho1—O4—C1	74.7 (7)
C21—C16—C17—C18	−1.0 (11)	Ho1^vi^—Ho1—O4—C1	13.0 (6)
C15—C16—C17—C18	−175.7 (6)	O7—C15—O6—Ho1	−2.2 (7)
C16—C17—C18—C19	−0.1 (11)	C16—C15—O6—Ho1	−179.0 (5)
C16—C17—C18—C22	178.2 (6)	O1^iv^—Ho1—O6—C15	141.6 (4)
C17—C18—C19—C20	4.0 (11)	O4—Ho1—O6—C15	−68.2 (4)
C22—C18—C19—C20	−174.3 (6)	O8^v^—Ho1—O6—C15	−142.5 (5)
C18—C19—C20—C21	−7.1 (10)	O3^vi^—Ho1—O6—C15	67.4 (4)
C18—C19—C20—N4	−178.6 (6)	O7^vi^—Ho1—O6—C15	−1.0 (5)
C17—C16—C21—C20	−1.9 (10)	O1W—Ho1—O6—C15	−142.5 (4)
C15—C16—C21—C20	172.8 (6)	O7—Ho1—O6—C15	1.1 (4)
C19—C20—C21—C16	6.1 (10)	Ho1^vi^—Ho1—O6—C15	0.1 (4)
N4—C20—C21—C16	177.8 (6)	O6—C15—O7—Ho1^vi^	168.5 (16)
C19—C18—C22—O9	−21.2 (10)	C16—C15—O7—Ho1^vi^	−15 (2)
C17—C18—C22—O9	160.5 (6)	Ho1—C15—O7—Ho1^vi^	167 (2)
C19—C18—C22—O8	158.1 (7)	O6—C15—O7—Ho1	2.0 (7)
C17—C18—C22—O8	−20.2 (10)	C16—C15—O7—Ho1	178.7 (6)
O10—C23—C24—C28	−143.0 (8)	O1^iv^—Ho1—O7—C15	−58.9 (4)
N4—C23—C24—C28	38.7 (10)	O4—Ho1—O7—C15	102.1 (4)
O10—C23—C24—C25	35.3 (10)	O8^v^—Ho1—O7—C15	36.7 (4)
N4—C23—C24—C25	−143.1 (6)	O3^vi^—Ho1—O7—C15	−105.2 (4)
C28—C24—C25—C26	1.3 (9)	O7^vi^—Ho1—O7—C15	177.1 (5)
C23—C24—C25—C26	−177.1 (6)	O1W—Ho1—O7—C15	140.0 (4)
C24—C25—C26—N3	0.8 (10)	O6—Ho1—O7—C15	−1.1 (4)
C25—C24—C28—C27	−4.9 (10)	Ho1^vi^—Ho1—O7—C15	177.1 (5)
C23—C24—C28—C27	173.4 (6)	O1^iv^—Ho1—O7—Ho1^vi^	124.0 (2)
N3—C27—C28—C24	6.5 (11)	O4—Ho1—O7—Ho1^vi^	−75.07 (18)
O7—C15—Ho1—O1^iv^	134.7 (4)	O8^v^—Ho1—O7—Ho1^vi^	−140.43 (17)
O6—C15—Ho1—O1^iv^	−43.2 (5)	O3^vi^—Ho1—O7—Ho1^vi^	77.65 (18)
O7—C15—Ho1—O4	−72.3 (4)	O7^vi^—Ho1—O7—Ho1^vi^	0.0
O6—C15—Ho1—O4	109.8 (4)	O1W—Ho1—O7—Ho1^vi^	−37.1 (4)
O7—C15—Ho1—O8^v^	−146.7 (4)	O6—Ho1—O7—Ho1^vi^	−178.3 (3)
O6—C15—Ho1—O8^v^	35.4 (4)	C15—Ho1—O7—Ho1^vi^	−177.1 (5)
O7—C15—Ho1—O3^vi^	68.1 (4)	O9—C22—O8—Ho1^v^	−30.4 (10)
O6—C15—Ho1—O3^vi^	−109.9 (4)	C18—C22—O8—Ho1^v^	150.3 (5)
O7—C15—Ho1—O7^vi^	−2.8 (5)		

Symmetry codes: (i) *x*+1, *y*+1, *z*−2; (ii) *x*+1, *y*+1, *z*−1; (iii) *x*−1, *y*−1, *z*+2; (iv) *x*, *y*+1, *z*; (v) −*x*, −*y*+2, −*z*+2; (vi) −*x*+1, −*y*+2, −*z*+2; (vii) *x*, *y*−1, *z*; (viii) *x*−1, *y*−1, *z*+1.

### Hydrogen-bond geometry (Å, º)

**Table:**

*D*—H···*A*	*D*—H	H···*A*	*D*···*A*	*D*—H···*A*
N2—H2···O3*W*^ix^	0.86	2.11	2.878 (9)	149
N4—H4···O5^vi^	0.86	2.08	2.925 (8)	168
O1*W*—H1*X*···O9^v^	0.85	2.05	2.562 (7)	118
O1*W*—H1*Y*···O10^x^	0.85	1.90	2.717 (7)	161
O2*W*—H2*X*···O3*W*^xi^	0.85	2.11	2.799 (8)	138
O2*W*—H2*Y*···O1*W*^xii^	0.85	2.34	3.136 (8)	156
O2*W*—H2*Y*···O9^xiii^	0.85	2.22	2.766 (8)	122
O3*W*—H3*X*···N2^xiv^	0.85	2.49	3.187 (9)	140
O3*W*—H3*Y*···O9^xv^	0.85	2.30	2.821 (7)	120

Symmetry codes: (v) −*x*, −*y*+2, −*z*+2; (vi) −*x*+1, −*y*+2, −*z*+2; (ix) *x*−1, *y*, *z*+1; (x) *x*, *y*, *z*+1; (xi) *x*−1, *y*, *z*; (xii) *x*, *y*−1, *z*−1; (xiii) −*x*, −*y*+1, −*z*+1; (xiv) −*x*+1, −*y*+1, −*z*+2; (xv) *x*+1, *y*−1, *z*.
